# Mechanical Characterization of a Novel Cyclic Olefin-Based Hot-Melt Adhesive

**DOI:** 10.3390/ma18040855

**Published:** 2025-02-15

**Authors:** Vasco C. M. B. Rodrigues, Ana T. F. Venâncio, Eduardo A. S. Marques, Ricardo J. C. Carbas, Armina Klein, Ejiri Kazuhiro, Björn Nelson, Lucas F. M. da Silva

**Affiliations:** 1 Institute of Science and Innovation in Mechanical and Industrial Engineering (INEGI), 4200-465 Porto, Portugal; avenancio@inegi.up.pt; 2 Department of Mechanical Engineering, Faculty of Engineering, University of Porto, 4169-007 Porto, Portugal; emarques@fe.up.pt (E.A.S.M.); lucas@fe.up.pt (L.F.M.d.S.); 3 Zeon Europe GmbH, Areal Böhler, Hansaallee 249, 40549 Düsseldorf, NRW, Germany

**Keywords:** cyclo-olefin-based polymer, adhesive bonding, adhesive characterization, hot-melt adhesive, fracture mechanics, automotive industry, electronic industry

## Abstract

A novel hot-melt cyclic olefin-based adhesive was designed as a transparent, non-tacky film of amorphous thermoplastic with a unique polymer micro-structure. The aim of the present paper is to assess the mechanical properties of the 0.1 mm thick COP hot-melt adhesive film through adhesive characterizations tests. The glass transition temperature was determined using dynamic mechanical analysis (DMA). For mechanical characterization, bulk and thick adherend shear specimens were manufactured and tested at a quasi-static rate, where at least three specimens were used to calculate the average and standard deviation values. Tensile tests revealed the effects of molecular chain drawing and reorientation before the onset of strain hardening. Thick adherend shear specimens were used to retrieve shear properties. Fracture behaviour was assessed with the double cantilever beam (DCB) test and end-notched flexure (ENF) test, for characterization under modes I and II, respectively. To study the in-joint behaviour, single lap joints (SLJs) of aluminium and carbon fibre-reinforced polymer (CFRP) were manufactured and tested under different temperatures. Results showed a progressive interfacial failure following adhesive plasticization, allowing deformation prior to failure at 8 MPa. An adhesive failure mode was confirmed through scanning electron microscopy (SEM) analysis of aluminium SLJ. The adhesive exhibits tensile properties comparable to existing adhesives, while demonstrating enhanced lap shear strength and a distinctive failure mechanism. These characteristics suggest potential advantages in applications involving heat and pressure across automotive, electronics and structural bonding sectors.

## 1. Introduction

Thermoplastic adhesives, commonly known as hot-melt adhesives (HMAs), are distinguished by their ability to soften upon heating, allowing easy separation [1,2]. Historically, solid wax has been used for temporary joints in various work materials. By melting the wax, materials can be joined, fixed and later separated by reheating. This method is particularly useful for materials such as glass, ceramics and silicones, which are non-magnetic and cannot be secured with a magnetic chuck. Despite being a natural and ancient material, the use of wax continues to grow due to its versatility. In electronics, several types of debondable adhesives are employed, particularly in the manufacturing of integrated circuit chips [3]. Initially, silicone ingots are affixed with an adhesive and sliced into wafers using a dicing saw. This adhesive can be softened by heating, allowing easy removal of any residue with a specific solvent or alkaline solution. Hot-melt adhesives have also found applications in housing construction, particularly for material recycling purposes. An example is the “ALLOVER Method” used by Japanese construction companies, which involves bonding wall or ceiling boards to beams using hot-melt tape and an induction heating machine [4]. Once in place, a heating coil will increase the temperature of at least one adherent conductor, securely joining the boards and beams. The weak tackiness of the tape simplifies the initial positioning. This bond is reversible, allowing the materials to be separated by reheating, facilitating recycling. The increasing use of hot-melt adhesives in various industries highlights their versatility and efficiency [2].

Among the innovations in HMA, there are very few types of hydrocarbon-based hot melt adhesives. This is because hydrocarbon polymers have generally been considered unsuitable as adhesives because of their crystalline nature, insolubility in solvents and low polarity. Cyclic olefins are unusual among hydrocarbon polymers in that they are amorphous and, therefore, soluble in certain solvents. When cyclic olefins are used as base polymers for olefin-based hot-melt adhesives, the new adhesives, unlike well-known hot-melt adhesives, have excellent transparency, impact resistance, tensile elongation, insulation properties and recyclability. Cyclic olefins can be categorised as cyclic olefin polymers (COPs), produced by ring-opening metathesis polymerisation (ROMP), or cyclic olefin copolymers (COCs), created via addition polymerisation [5,6,7]. Compared to conventional adhesives, this new adhesive offers significantly improved adhesion, flexibility and durability to a wide range of substrates, including metals, plastics and composite materials. This versatility is particularly valuable in industries that require reliable and robust adhesive solutions. In the automotive sector, this novel adhesive is being considered for the assembly of battery packs, interior components and structural parts of electric vehicles (EVs) [8]. In addition, its low water absorption and lack of hydrolysis can contribute to the longevity and safety of automotive components. In the electronics industry, this new adhesive is also expected to play a role, especially in sensitive components, such as integrated circuits, display screens and optical equipment. Furthermore, the ability to form strong yet peelable bonds is in line with the industry’s drive for sustainability by facilitating repair and recycling. In the construction industry, this novel adhesive provides a cooperative and durable bond to a variety of building materials, and its ability to withstand environmental conditions, such as extreme temperatures, can extend the life of structural components. Furthermore, the reversible nature of HMA supports the recycling and reuse of building materials, contributing to environmentally friendly construction methods [9,10].

This work aims to study the potential of a novel 0.1 mm thick cyclic olefin-based adhesive film, a development product of Zeon^®^ Corporation (Chiyoda-ku, Tokyo, Japan) named LS adhesive. In this work, rheological and mechanical properties were determined using adhesive characterisation tests as a first systematic approach to characterising this type of adhesive. Dynamic mechanical analysis (DMA) was performed to assess the relaxation of the material and retrieve the glass transition temperature $T_{g}$. To comprehensively evaluate the mechanical properties of the hot-melt cyclic olefin-based network adhesive for the first time, a diverse array of standard and non-standard test methodologies were implemented under quasi-static loading conditions. Tensile properties are determined following bulk tensile properties and shear properties are determined following the thick adherent shear test (TAST). Regarding the fracture toughness of the material, researchers have consistently used the Double Cantilever Beam (DCB) test to determine the critical energy release rate in Mode I and the end-notched flexure (ENF) test for Mode II [3,11,12]. The use of the J-integral method provides precision in estimating the fracture toughness values for both modes [13,14]. Subsequently, similar and dissimilar single lap joints (SLJs) using aluminium and carbon fibre-reinforced polymer adherents (CFRPs) were manufactured to analyse the failure mode of the joint and understand the adhesion for different material substrates at room temperature and high temperature. The reported property values were determined using a minimum of three specimens to calculate the average and respective standard deviation.

## 2. Experimental Details

### 2.1. Materials

The 0.1mm thick cyclic olefin-based adhesive film (LS adhesive) was provided by Zeon^®^ Corporation (Chiyoda-ku, Tokyo, Japan). This material was reported to have a softening temperature of 122 °C. For all experiments performed, the material was subjected to a 175 °C adhesion stage on a hot press at 0.8 MPa for 45 min, allowing it to flow and wet the surface. The cooling stage was gradually carried out at ambient temperature, with the press heating system turned off.

### 2.2. Specimen Manufacturing

#### 2.2.1. Bulk Specimens

**Figure 1:**
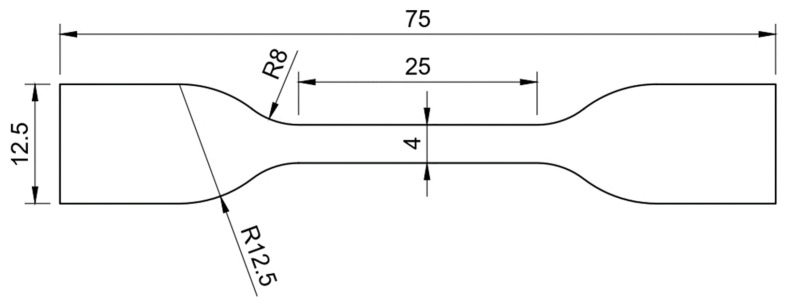
Bulk specimen dimensions according to DIN 53504 standard (dimensions in mm).

The bulk test provides the tensile properties of the film adhesive, determining the Young’s modulus and tensile strength. The stress–strain curve provides the elastic and plastic behaviour of the material [11]. Six bulk dumbbell specimens (also known as dogbone) for LS were manufactured according to the DIN 53504 standard [15,16]. The specimens used to describe the tensile behaviour of the film adhesive are schematically represented in Figure 1. Due to the fact that the material was provided in sheets with a predetermined thickness, the thickness deviated from the standard and corresponded to 0.1 mm.

The hot-melt film sheets had to be cut and trimmed with precision to follow the standard dimensions. To do this, a computer-aided design (CAD) model was made and a 3D-printed polylactide (PLA) part served as an exact cutting guide when cutting out the bulk specimens [17]. Thereafter, the 3D-printed part was pressed against the film protector paper layer and the film cut with a medical 10 mm scalpel. After the desired geometry was achieved, aluminium tabs were added to the ends of the specimens using a pressure sensitive adhesive (PSA), given the non-tackiness of the hot-melt film. This procedure was necessary to secure the specimens to the gripping system of the testing machine.

#### 2.2.2. Thick Adherend Shear Specimens

**Figure 2:**
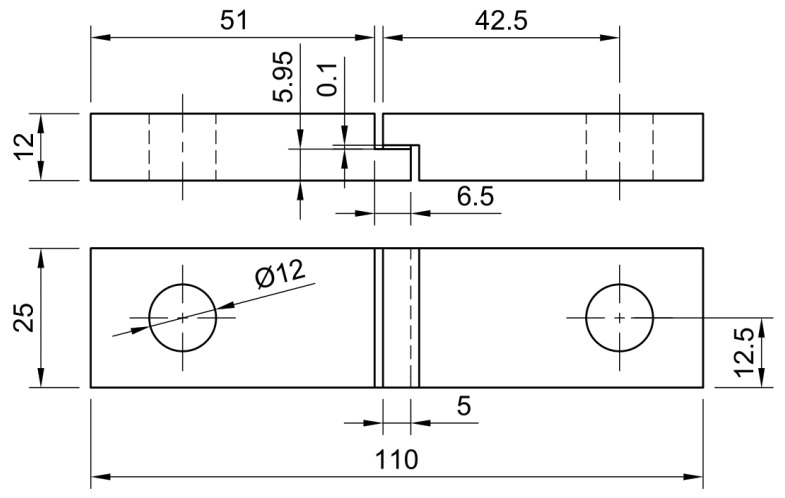
Thick adherend shear joint (dimensions in mm).

TAST aluminium specimens were manufactured and precisely rectified to ensure an adhesive thickness corresponding to the thickness of the HMA film, according to the ISO 11003-2 standard [18]. Figure 2 illustrates the dimensions of the specimens with a minor modification from standard measures. The height of the bonding section was increased from 5.75 mm to 5.95 mm to ensure that the final thickness exactly matched the HMA film thickness. This slight adjustment allows for a more accurate representation of the adhesive’s performance under shear conditions while maintaining overall compliance with the standardised testing method, avoiding the need for layer stacking. At least three TAST specimens were tested to ensure consistent shear values [19].

#### 2.2.3. Double Cantilever Beam Specimens

The double cantilever beam (DCB) test is based on a pure crack opening in Mode I and aims to characterise the fracture toughness in the respective mode. The DCB test was performed according to the ASTM D3433 standards [20], as well as ISO 25217 [21]. The specimen consists of two 4 mm thick CFRP substrates, where a layer of the film adhesive is applied between them. The substrates were manufactured to be 25 by 295 mm. Thereafter, they were cleaned and plasma-treated, to ensure a better adhesion. The DCBs were placed in a steel mould where the hot-melt film was inserted.

**Figure 3:**
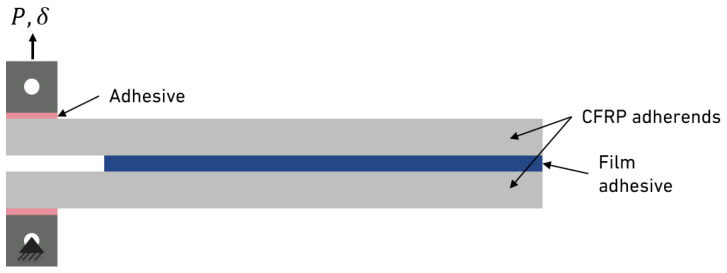
CFRP DCB specimens with loading blocks.

After cooling, steel loading blocks (20 by 20 by 20 mm cubes) were bonded to one edge of the CFRP upper and lower adherents using a modified two-component (2 k) epoxy adhesive. For that, the blocks were sandblasted and degreased with acetone, while the DCB substrates were grit-sanded prior to acetone cleaning. An initial crack length was established at 45 mm. Figure 3 displays the geometry of the DCB specimen, following the previous standards. At least three DCB specimens were tested to ensure consistent energy release rate values.

#### 2.2.4. Single Lap Joints

**Figure 4:**
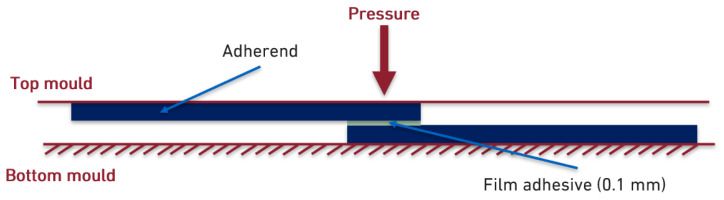
Manufacturing scheme of SLJs.

To study the performance of the hot-melt adhesive in a joint, aluminium 6082-T6 and CFRP (SEAL^®^ Texipreg HS 160 RM) SLJs were manufactured using only one overlap length of 12.5 mm. The joints were produced in an aluminium mould (Figure 4) coated with a mould release agent.

The aluminium alloy 6082-T6 substrates with a thickness of 2 mm were machined to 25 by 107.5 mm. The CFRP adherends manufactured had a thickness of 2.1 mm.

### 2.3. Testing Procedures

#### 2.3.1. Dynamic Mechanical Analysis

The glass transition temperature was determined using DMA conducted with a Netzsch DMA 242 E Artemis (Erich Netzsch GmbH & Co. Holding KG, Selb, Germany) testing machine. A rectangular sample with dimensions of 12 by 6 by 0.1 mm was prepared for testing in tensile deformation mode. The analysis was carried out at a frequency of 1 Hz with a constant displacement amplitude of 40 µm, in a temperature range of 24 °C to 140 °C with a heating rate of 2 °C/min.

#### 2.3.2. Bulk Tensile Test

Bulk tensile tests were performed using a Mecmesin^®^ MultiTest-10i twin-column tensile tester (West Sussex, UK), equipped with a 10 Newton load cell, at constant crosshead speed of 10 mm/min. Stress–strain curves were recorded for each test until the failure of each specimen. Before each test, all dimensions of the specimen were measured to ensure the precision of the tensile stress measurements. The specimens were secured to the machine using clamps that held both ends of the specimen through aluminium tabs that were attached prior to the test. Since the calculation of engineering stress and strain values assumes a constant cross-sectional area throughout the test, true stress and strain values were also obtained. This assessment allowed to evaluate the range within which this assumption is valid, according to the following equations:

(1) $$\sigma_{true}=\sigma \left(1+\epsilon_{eng}\right)$$

(2) $$\epsilon_{true}=ln\left(1+\epsilon_{eng}\right)$$

where σ represents the engineering stress and $\epsilon_{eng}$ denotes the engineering strain. The engineering stress was calculated by dividing the load by the initial cross-sectional area. The nominal strain was determined by considering the deformation as the displacement of the machine divided by the original gauge length of the bulk specimen, $L_{0}$. Since this approach is not the most adequate to determine the engineering strain, digital image correlation (DIC) was used to output the strain field of the initial part of the test, corresponding to the elastic regime. The bulk specimens were coated with matte white polyurethane-based spray paint and then speckled with black ink to create a suitable pattern. The images were captured with a digital camera (Nikon^®^ D5300) and treated in GOM Correlate^®^ software (version 2019 Hotfix 7, Rev. 128764). In total, six specimens were tested to ensure decent test repeatability.

#### 2.3.3. Thick Adherend Shear Test

TAST specimens were tested at a quasi-static crosshead rate of 1 mm/min on an INSTRON^®^ 3367 (Illinois Tool Works, Hopkinton, MA, USA) universal testing machine, equipped with a 30 kN load cell. Two methods were used for strain measurements: a high-resolution extensometer and DIC [11]. Both shear strain calculations can be outputted as

(3) $$\gamma_{s_{Extensometer}}=\frac{\epsilon_{extensometer}\cdot L_{0}}{t}$$

(4) $$\gamma_{s_{DIC}}=\frac{\Delta L_{DIC}}{t}$$

The shear stress $\tau_{s}$ is calculated by dividing the maximum load by the bond area (5 by 25 mm). For an isotropic material, where the relationship E=2G(1+ν) is valid, the shear modulus *G* can be determined from the slope of the linear elastic region in the shear stress–strain curve. At least three specimens values were enough to output the material shear properties.

#### 2.3.4. Double Cantilever Beam Test

For DCB specimen testing, the same INSTRON^®^ 3367 universal testing machine (Illinois Tool Works, Hopkinton, MA, USA) was used. The specimens were assembled on the test machine by connecting the loading blocks to the machine attachment elements with stiff steel pins. All tests were carried out in displacement-controlled mode at a constant crosshead speed of 2 mm/min. To facilitate the calculation of fracture energy, rotations at the loading points were recorded using a set of Sick^®^ TMS22E-PKH080 inertial (Waldkirch, Germany) sensors acting as inclinometers, which were synchronized with the universal testing machine. The loads and angles measured during the tests were used to apply the J-integral approach to determine the fracture energy for each test. For Mode I loading, the value of *J* for a rectangular section double cantilever beam specimen is given by [13]

(5) $$J_{I}=\int_{\Gamma }\left(Wdy-T_{i}\frac{\partial u_{i}}{\partial x}ds)\right)$$

where the specimen’s outer boundary serves as the contour of integration for the sake of this analysis. It is therefore seen that, everywhere except at the load points, contributions to this integral are zero (so long as the load sites and crack tip are positioned remotely). If the traction σ remains constant throughout the dx increment,

(6) $$T_{i}=\sigma =\frac{P}{dx}=c^{te}$$

Since ds corresponds to dx or minus dx in the lower and upper contour, respectively, the *J* integral can be evaluated as

(7) $$J_{I}=P\left[{\left(\frac{\partial u_{y}}{\partial x}\right)}_{bottom}-{\left(\frac{\partial u_{y}}{\partial x}\right)}_{top}\right]$$

Having the expression above adapted for the real configuration in which the sensor rotation data are inputted, the *J* value proposed by Paris and Paris [13] is expressed as

(8) $$J_{I}=\frac{P}{b}\left(\theta_{top}-\theta_{bottom}\right)$$

where *P* is the applied load, *b* is the width of the specimen, and $\theta_{up}$ and $\theta_{low}$ are the relative rotations of the upper and lower substrates at the loading points, respectively. The difference between these angles is named the total rotation ($\theta_{total}$) [22]. This relationship holds true, considering the path independence property of the J-integral for both linear and non-linear elastic materials and assuming that the region outside the fracture process zone (FPZ) remains elastic during fracture. For DCB tests, the analysis of at least three specimens was performed to ensure the repeatability of the results.

#### 2.3.5. End-Notched Flexure Tests

**Figure 5:**
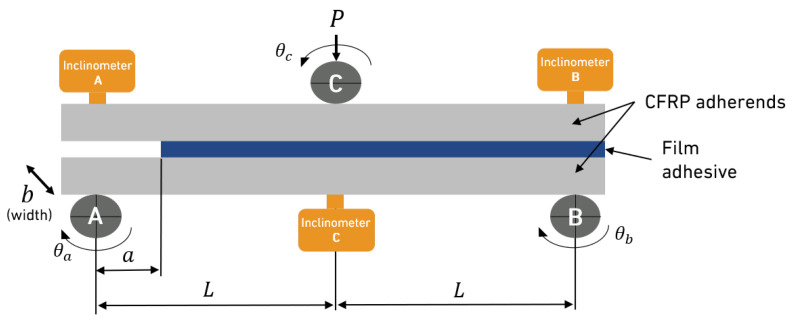
ENF test setup.

The ENF test was performed using DCB specimens without loading blocks. Three of the aforementioned inclinometers were positioned as shown in Figure 5, according to the loading points. The initial crack length (*a*) was 50 mm and *L* was 140 mm. The test was performed at a constant crosshead speed of 0.5 mm/min.

The J-integral control volume approach is valid for ENF specimens. Provided an arbitrary contour Γ surrounding the crack tip, the J-Integral is given by

(9) $$J=\int_{\Gamma }\left(w\;dy-T_{i}\frac{\partial u_{i}}{\partial x}\;ds\right)$$

where $u_{i}$ contains the components of the displacement vector and ds is an infinitesimal length increase along the path Γ. The strain energy density (*w*) is

(10) $$w=\int_{0}^{\epsilon_{ij}}\sigma_{ij}d\epsilon_{ij}$$

From Cauchy’s theorem, $T_{i}=\sigma_{ij}n_{j}$, where $T_{i}$ corresponds to the traction along the path, $\sigma_{ij}$ is the stress tensor and $n_{j}$ the unit components of the normal volume control vector Γ. $u_{i}$ contains the components of the displacement vector and ds is an infinitesimal length increase along the Γ path [23].

The *J* decomposition for the ENF specimens can be given by an independent crack length equation [14,23,24,25,26], as follows:

(11) $$J_{II}=\frac{P}{2b}\left[\theta_{A}+\theta_{B}-2\theta_{C}\right]$$

However, by employing linear elastic fracture mechanics (LEFM), the relative slopes between two positions are given by analytical equations, which, as a summary, result in [23,27]

(12) $$J_{II}=G_{II}=\frac{9P^{2}a^{2}}{16E_{11}b^{2}h^{3}}$$

Three ENF specimens were tested under pure Mode II condition.

#### 2.3.6. Single Lap Joint Test

For single lap joint testing, the aforementioned INSTRON^®^ 3367 universal testing machine (Illinois Tool Works, Hopkinton, MA, USA) was used. All tests were carried out in displacement-controlled mode at a constant crosshead speed of 1 mm/min. A high-speed camera, Kron Technologies^®^ Chronos 1.4 (Eastlake Drive, BC, Canada) was used to record the lap shear joint test to improve the analysis of the load–displacement curve. Four lap shear joints were tested for each condition to ensure a consistent lap shear strength.

#### 2.3.7. Scanning Electron Microscopy Analysis

Scanning electron microscopy (SEM) was performed to analyse the failure mode of aluminium SLJs. A high-resolution (Schottky) environmental scanning electron microscope (FEI Quanta 400 FEG ESEM (FEI Company, Hillsboro, OR, USA)) equipped with X-ray microanalysis and electron backscatter diffraction (EDAX Genesis X4M (FEI Company, Hillsboro, OR, USA)) was used to perform scanning electron microscopy and energy dispersion X-ray spectroscopy (EDS) analysis.

## 3. Results

### 3.1. Rheological Properties

**Figure 6:**
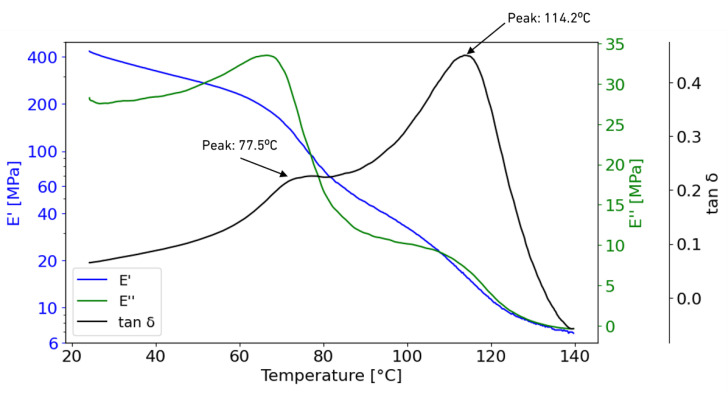
DMA results highlighting the β-relaxation and glass transition temperature.

Figure 6 shows the elastic ($E^{\prime }$) and loss modulus ($E^{\prime \prime }$) as well as the calculated $T_{g}$ of the LS film. This parameter is determined by dividing the viscous component by the elastic response [28,29]. LS has a $T_{g}$ of 114.2 °C, which is common for these types of formulations [5]. The film also shows a smooth β-relaxation ($T_{\beta }$) at 77.5 °C, a secondary relaxation which is generally attributed to branching chains or side groups, resulting from localised molecular motions within the polymer structure [29,30].

### 3.2. Bulk Tensile Tests

**Figure 7:**
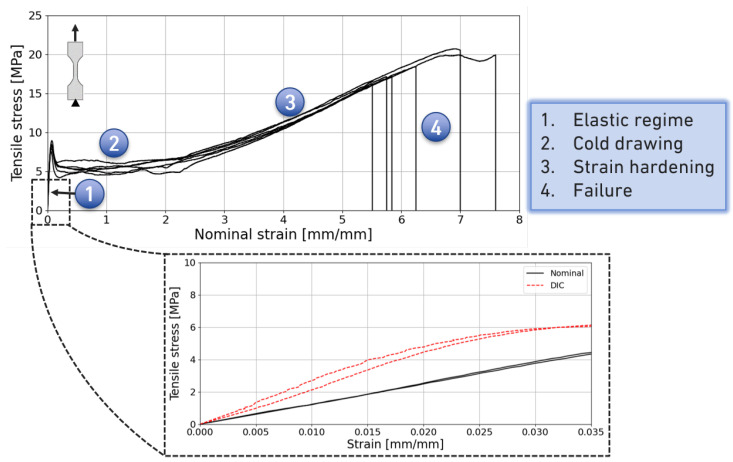
Stress–strain engineering curves of the six bulk specimens tested.

The characteristic stress–strain curves for a crosshead speed of 10 mm/min are presented in Figure 7. During the test, the separation of four stages is evident due to the elastic regime, the cold drawing of the molecular chains and chain reorganisation, the strain hardening due to the chain alignment, and failure. This deformation mechanism is a typical phenomenon for thermoplastic materials.

**Figure 8:**
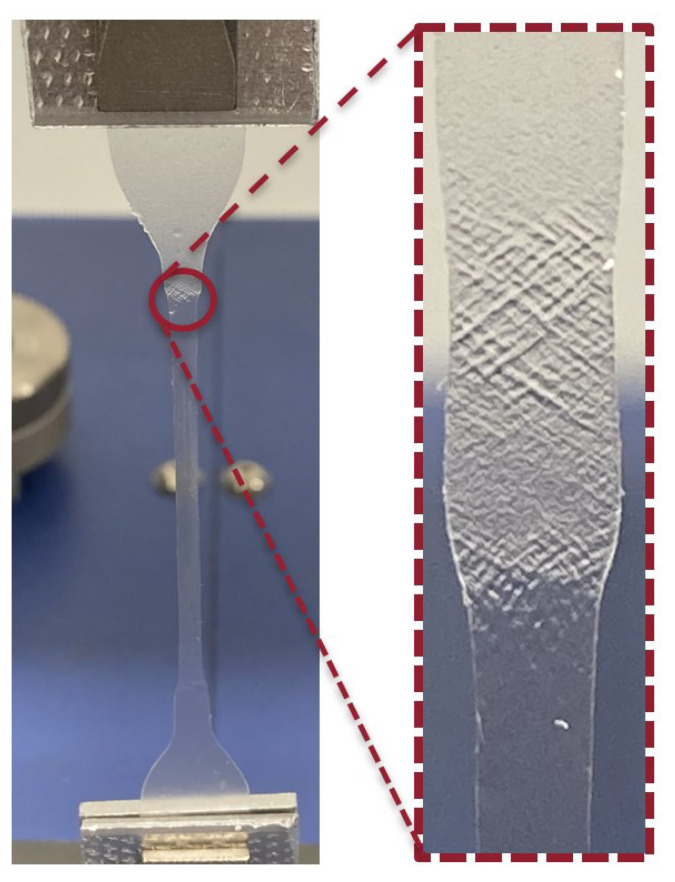
Cold drawing phenomenon observed during the tensile test.

Figure 8 displays a photograph captured during the chain reorganisation stage, where the aligned section is much more transparent, highlighting a zoomed-in section that is still undergoing chain alignment.

The elastic properties outputted provided a Young’s modulus (*E*) of 243.5±30.0 MPa, a yield strength ($\sigma_{y}$) of 8.24±0.43 MPa, tensile strength ($\sigma_{f}$) of 18.3±1.6 MPa and an elongation ($\epsilon_{f}$) of 630±70%. There are dissimilarities regarding the output values of Young’s moduli between tensile tests in plastic materials standards (ASTM D638 [31], ASTM D412 [32] and ISO 527-1 [33]); nonetheless, it can be considered as the slope value from a straight line starting in zero up to a point where it no longer overlaps with the stress–strain curve. In fact, Figure 7 shows two graphs, one being the stress versus nominal strain curve and the other the stress–strain zoom-in of the elastic regime.

**Figure 9:**
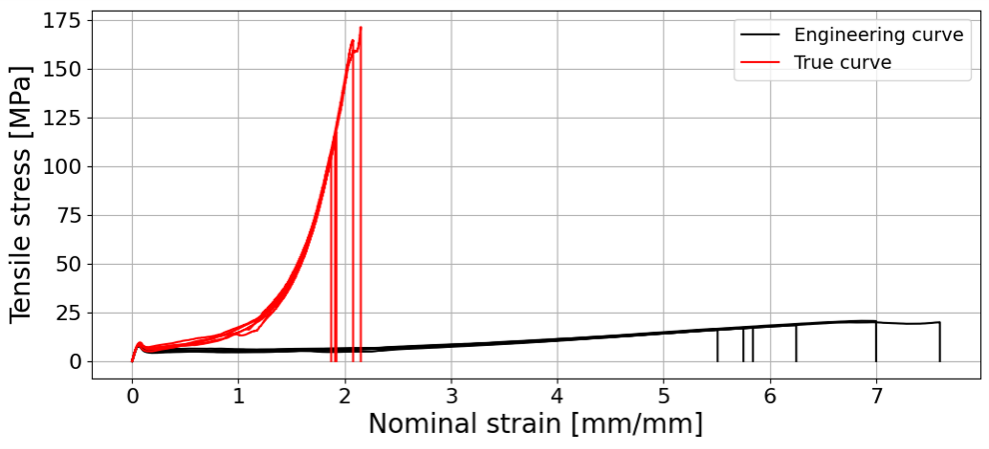
Engineering and true stress–nominal strain curve.

The nominal strain computed during the test was an approximation, as it was not feasible to record the true strain throughout the experiment. The nominal strain was calculated by dividing the crosshead displacement of the testing machine by 25 mm, which corresponds to the length of the central portion of the dog-bone specimen, where the cross-section is reduced. However, due to the high deformation experienced by the specimen and the distortion of the speckle pattern, the DIC methodology was unable to capture the full strain field throughout the duration of the testing. Despite this limitation, DIC analysis offers a more accurate measurement of strain compared to machine displacement data to calculate the nominal strain. This hypothesis stems from the assumption that the specimen deforms exclusively within the 25 mm gauge length, when in reality the deformation extends up to the edges of the dog-bone specimen. This behaviour becomes apparent after the second stage of deformation, as depicted in Figure 7. At this point, the pre-aligned molecular chains within the 4 mm wide section of the specimen demonstrate greater strength than the wider regions near the coupon edges. During the third stage, characterised by a strain hardening phenomenon, cold drawing continues to occur, particularly in the broader sections of the specimen. Consequently, the Young’s modulus was estimated using the strain values derived from the DIC analysis, ensuring a more precise determination of the elastic properties of the material. Having the stress–nominal strain curve, the true stress and the true nominal strain can be calculated, as shown in Figure 9.

### 3.3. Thick Adherend Shear Tests

**Figure 10:**
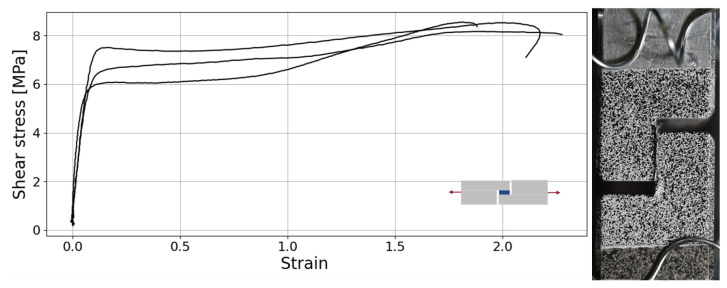
Shear stress–strain curve (**left**) and TAST specimen speckled for DIC analysis and with spring clippers to fix the extensometer (**right**).

The TAST was able to output the adhesive shear properties. The shear strain depicted in the shear stress–strain curve, as illustrated in Figure 10, was derived from a linear extensometer. The use of displacement linear variable differential transformer (LVDT) yielded more reliable measurements throughout the tests, exhibiting a lower signal-to-noise ratio compared to DIC. The improved reliability stems from the method’s ability to detect minute relative movements between adherents, which is particularly advantageous when dealing with very thin adhesive layers. Assuming a Poisson ratio of 0.4, the shear modulus (*G*) is 90 MPa, the shear strength ($\tau_{f}$) 8.45±0.12 MPa and the maximum shear strain ($\gamma_{f}$) is 201±23%. The three specimens tested showed adhesive failure.

### 3.4. Double Cantilever Beam Tests

Although steel DCB substrates were initially machined and tested, their standardised thickness of 12.7 mm resulted in minimal rotation during testing. This lack of rotation caused the sensors to record excessive noise. To address this issue, thinner substrates were used. CFRP was chosen because of its ease of fabrication and practical advantages. Its use allowed for greater rotation, which reduced the noise detected by the sensors. Given that there was good adhesion to the plasma-treated CFRP in comparison to degreased aluminium (mentioned in the next section) and the volume control data reduction scheme, the use of more elastic substrates would not influence the final result. This approach represented the most effective engineering solution to accurately determine the critical energy release rate in Mode I at the interface [13,34].

**Figure 11:**
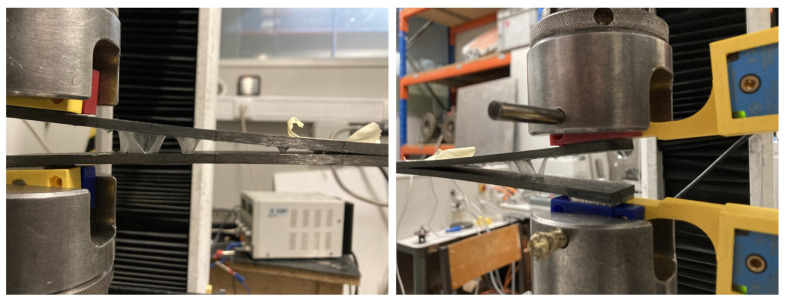
Reported stretching phenomenon during DCB tests.

During testing, the hot-melt film detached interfacially from both substrates, leading to an irregular, non-linear crack front and propagation. This detachment ahead of the crack front introduced challenges in accurately measuring the critical energy release rate, an issue that has not been widely reported in fracture energy measurements for interfacial delamination specimens [35]. In addition, a stretching phenomenon was observed in all specimens tested, as illustrated in Figure 11. This provided incorrect measurements of the interfacial value of $G_{I}$, since the Mode I DCB test propagated the crack by the detached interface along with the tension load of the unattached adhesive film.

**Figure 12:**
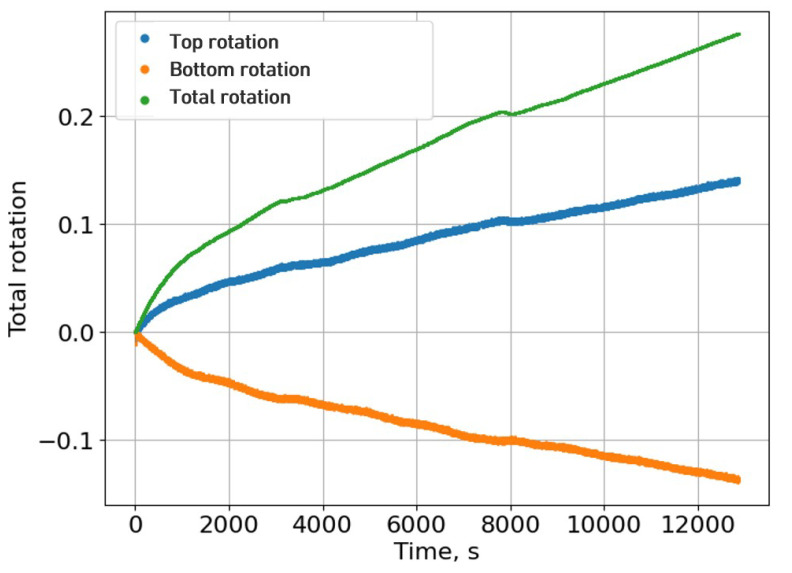
Rotations measured with the sensors and the total rotation curve.

**Figure 13:**
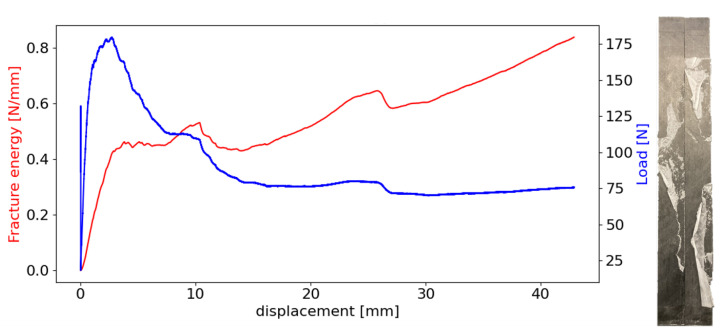
P-delta and R-curves with a representative opened DCB specimen.

Using the J-integral method as the data reduction scheme to withdraw the energy release rate as a function of the machine displacement, it was evident that no plateau was present in the R-curve of the energy values. According to Equation (8), the *J* value is given by multiplying the measured load by the total rotation. In a real scenario, during crack propagation, the load would decrease as the crack advances as the total rotation increases, having an almost constant value of energy, forming a plateau in the R-curve. However, provided that the stretching phenomenon is taken into account, it becomes clear that the expected downward trend in the P-delta curve is interrupted because of the tension resistance of the detached adhesive film. As a result, since the total rotation follows an upward trend (as shown in Figure 12), the fracture energy continuously increases with the opening displacement, rather than reaching the anticipated plateau in the R-curve. This was interestingly not observed for the crack blunting at the beginning of the test, where there was a small plateau. Upon reviewing video footage of the test from the digital camera, there was no reported stretching at the first 5 millimetres of crack propagation; therefore, the initial plateau value was considered as the average critical energy release rate of the film adhesive and a plasma-treated CFRP interface of 0.41±0.02 N/mm (Figure 13). The average result corresponds to the three best DCB specimens tested. Figure 13 shows a graph of the P-delta curve of one of the specimens tested with the output fracture energy as a function of machine displacement and the typical fracture surface of the opened CFRP DCB specimen.

### 3.5. End-Notched Flexure Tests

**Figure 14:**
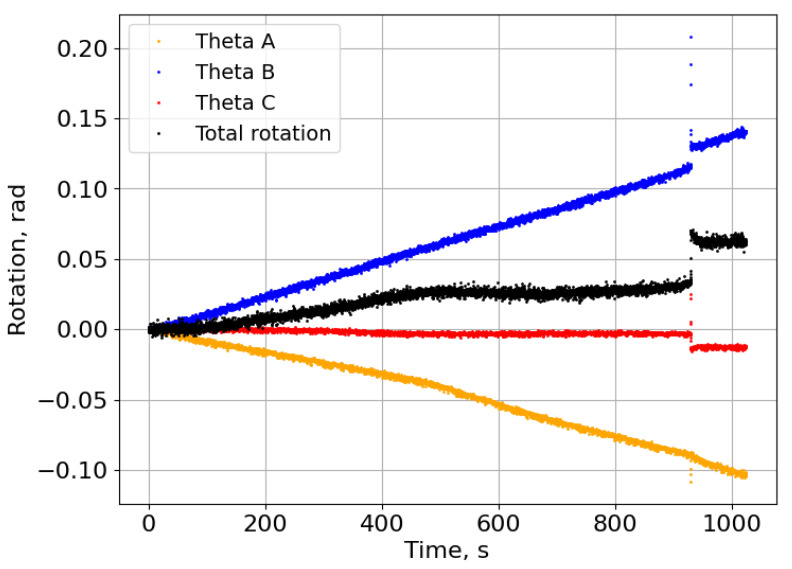
Rotations measured during one of the tested ENF specimens.

**Figure 15:**
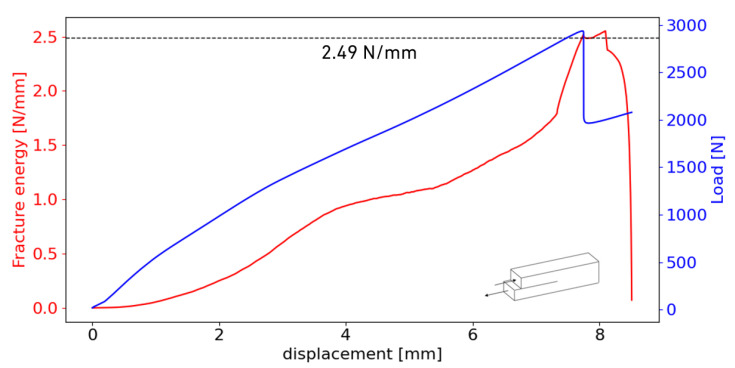
P-δ and fracture energy curve of the ENF specimen with the rotations displayed in Figure 14.

Figure 14 shows the rotations measured in one of the tested specimens. The propagation of the crack was sudden, similar to that of brittle adhesives [27]. This behaviour is evident in two key observations: the rapid change in rotations and the precipitous drop in the load–displacement curve, clearly illustrated in Figure 15. This figure also shows the relationship between fracture energy and displacement, highlighting the presence of a plateau immediately after the drop in the load–displacement curve. This plateau indicates a stabilisation in fracture energy, suggesting a consistent energy absorption phase after initial crack propagation. An average value of 2.57±0.09 N/mm was found to be the critical energy release rate of the film adhesive under pure Mode II loading. This value corresponds to the three best ENF specimens tested.

### 3.6. Single Lap Joint Tests

**Figure 16:**
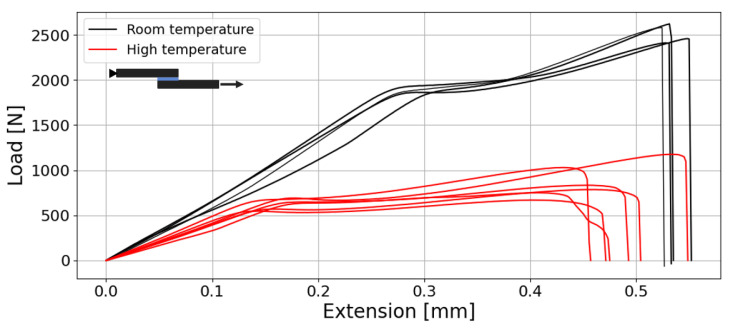
Lap shear curves for aluminium SLJs tested at room and high (80 °C) temperature.

All test sets took at least four specimens for acceptable repeatability. The load–displacement curves for the aluminium SLJs tested at room temperature (23 °C) and high temperature (80 °C) are shown in Figure 16.

**Figure 17:**
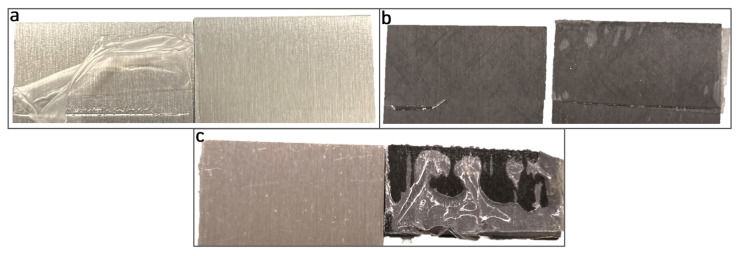
Typical failure mode for SLJ using (**a**) similar aluminium substrates; (**b**) similar plasma-treated CFRP substrates; (**c**) dissimilar Al-CFRP substrates.

It is evident that there is a linear elastic behaviour from the joint up to 2 kN, where, beyond this load value, the adhesive begins to yield, enhancing its strength. The failure is not sudden, providing a warning prior to the rupture of the joint. What was interesting to note during lap shear testing was the failure surface, which is always adhesive, as the DCB specimens, as shown in Figure 17 for similar joints (aluminium and CFRP) and dissimilar Al-CFRP joints, which show curve types that are very similar to the aluminium joints tested at room temperature. For high-temperature testing, two conclusions can be drawn: the first is the loss in lap shear strength (LSS) from 8 to 2.7 MPa and the second is the earlier yielding stage of the adhesive, despite the similar elongation to failure value.

**Figure 18:**
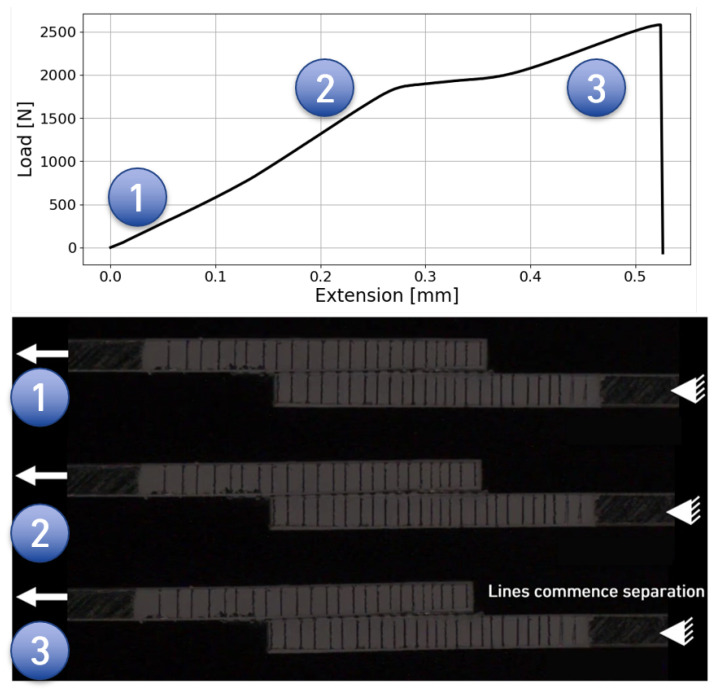
Al SLJ recording during the test.

In order to confirm the linear elastic behaviour and material yield, a high-speed camera was used to record one of the similar aluminium SLJ. The specimen was coated with white mate paint, in which straight black lines were drawn using a 0.3 mm ball pen. In Figure 18, it is possible to see that, at first, the lines are aligned and the specimen does not suffer from any bending phenomenon since the registered load is low. Thereafter, at the second stage, almost to the end of the linear regime, the black lines remain aligned despite some tilting that is observed, which is common for SLJ testing [3]. Finally, after the elastic peak is reached, the plastic domain of the adhesive layer appears clearly, proved by the line separation.

The lap shear strength values determined from the lap shear tests were 8.06±0.28 MPa for the acetone-degreased aluminium joints, 5.66±0.33 MPa for the acetone-degreased CFRP, 8.46±0.65 MPa for the plasma-treated CFRP joints and 7.55±0.19 MPa for the dissimilar joints. Aluminium SLJs tested at 80 °C showed an LSS of 2.69±0.40 MPa, showing one third of the LSS compared to the room temperature test. All lap shear joints showed similar P-delta curves. The increased LSS of the plasma treatment CFRP specimens allowed to clearly increase the level of adhesion and reach a performance matching that of the aluminium joints.

### 3.7. Failure Mode Analysis of Lap Shear Joints

**Figure 19:**
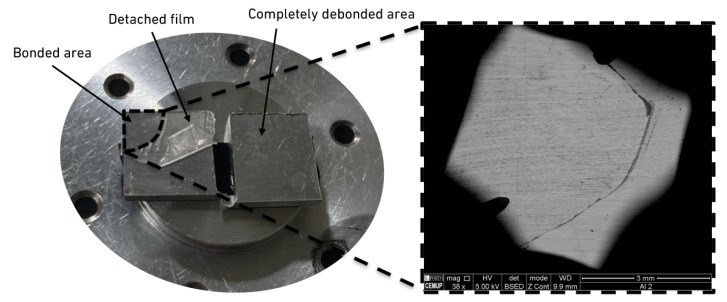
Aluminium SLJ sample and detail of bonded film (left) and correspondent BSED image from SEM.

The failure of the lap shear and DCB tests was similar, showing that both adhesive failures (AFs) are visible to the naked eye. To ensure this, aluminium adherents from SLJ tests were analysed in SEM [36,37,38]. Figure 19 shows the surface of samples from one tested lap shear specimen with some film still attached and the SEM backscattered electron detector (BSED) image of the corresponding surfaces.

**Figure 20:**
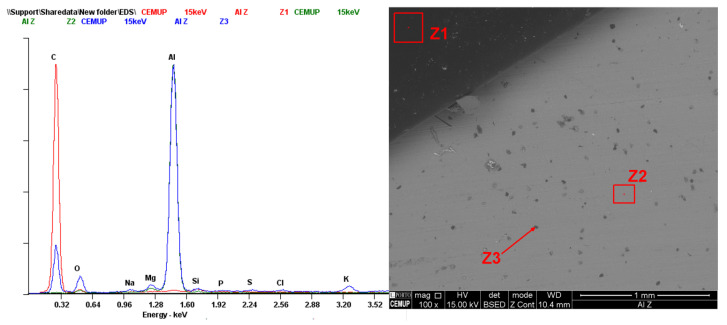
EDS performed at 15 KV on the adhesive film (Z1), aluminium surface (Z2) and a residue present in the adherent surface (Z3).

Dispersive X-ray spectroscopy was performed to see the spectrum of both the debonded area and the HMA. Having this, it is possible to ensure that the spectre of any residue present in the adherent surface has a matching spectrum with the adhesive film. Figure 20 shows an EDS analysis in the left specimen of Figure 19. The spectrum of the adhesive film (Z1) revealed a high concentration of carbon (C), as expected. In contrast, the adherent surface (Z2) exhibited a significant presence of aluminium (Al), with lower proportions of carbon and oxygen (O) compared to the film. The residue (Z3) on the adherent surface also showed a substantial amount of Al, even when analysed at 5 kV, similarly lacking proportional amounts of C and O. This shows that no residue of the adhesive film was left on the adherent surface.

**Figure 21:**
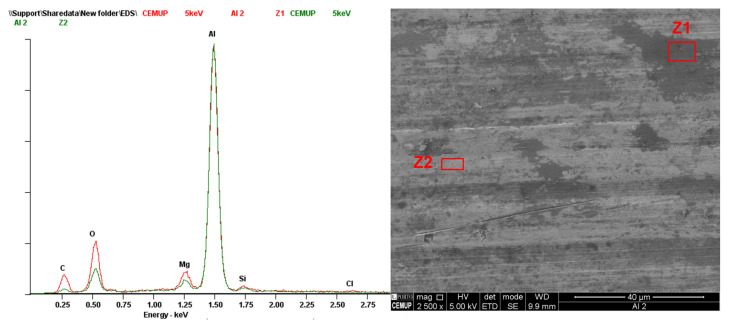
EDS performed at 5 KV on the aluminium surface after the peeling of the adhesive film.

To validate this assumption, the bonded adhesive film was then hand-peeled and the aluminium surface was immediately analysed, ensuring that no contamination occurred. The results are shown in Figure 21, highlighting dark grey (Z1) and light grey (Z2) areas. Both show a similar spectrum, with the darker area having a greater quantity of C and O, hypothetically due to aluminium oxidation or corrosion. However, they are not similar enough to the adhesive spectrum and guarantee a cohesive failure (CF) of the joint.

## 4. Discussion

Table 1 summarises the mechanical properties obtained from the mechanical characterisation tests, while Table 2 agglomerates the lap shear tests results.

Cyclic olefins enhance HMAs by introducing rigid ring structures that provide superior mechanical properties compared to standard thermoplastic polymers [5,6]. Having the material ID card of the LS adhesive, it is interesting to benchmark with other types of COCs. Studies found in the literature commonly report two mechanical characterisation strength tests, namely bulk tensile tests [15,39,40,41,42,43] and lap shear tests [44,45,46].

LS has tensile properties similar to those of common HMAs recently reported in the literature, agglomerated in Table 3. Zhang et al. [39] studied the influence of combining different percentages of a COC with linear low-density polyethylene (LLDPE) in measuring the dielectric constant; nevertheless, they also performed tensile tests. The concentration of COCs changes the mechanical properties, reducing elongation and increasing tensile strength with a similar stiffness value. Han et al. [42] aimed to develop a method to create circular olefin copolymers that could be synthesised from ethylene and α-olefins, thus allowing a closed-loop lifecycle for these materials. The recyclable olefin random copolymer (rPO) exhibited a stress–strain curve closely resembling that of the adhesive analysed in this study. The bulk tensile properties of the circular olefin copolymers indicate their potential as effective alternatives to traditional polyethylene (PE) materials, offering the added advantage of recyclability. Similar properties were also found in Sun et al. [47], which have synthesised and characterised a polyester block polyamide hot-melt adhesive (PPHMA), Kose et al. [40], which combined COC compounds with different concentrations of aluminium powder and Kricho et al. [41], who studied a commercial polyolefin-based HMA. An elastomeric thermoplastic HMA, characterised by its lower stiffness and strength but exceptional elongation, is also agglomerated [43].

Lap shear tests revealed an adhesion strength of 8 MPa for aluminium adherents, achieving 8.5 MPa with plasma-treated CFRP substrates. The failure is not sudden; it shows an area with a plastic yield of the HMA prior to the adhesive failure. The range of lap shear strength values is in accordance with paste-based semi-structural adhesives [19,48,49,50]. Table 4 benchmarks the lap shear values of similar materials reported in the literature. It is important to mention that most lap shear tests have been reported to fail adhesively when using supramolecular epoxy hot-melt adhesives (SEHMAs) [51] and polyamide hot-melt adhesive (PAHMA) [52]. Parker et al. [53] tested SLJs using stainless steel at different temperatures and strain rates to study LSS and failure mode of ethylene vinyl acetate (EVA) copolymers mixed with aromatic hydrocarbon resin HMA. They reported a constant adhesion failure at room temperature, regardless of the strain rate. However, when the temperature increased to 70 °C, the failure mode changed from AF to CF also with increasing strain rate. Hence, under impact conditions, the joint would fail cohesively in the adhesive. Other studies have also reported failure of the interface rather than CF in the adhesive [51,52,54].

As reported by Zhang et al. [39], the addition of COCs to an HMA increases its overall stiffness and strength but reduces elongation. Topas^®^ is renowned for its cyclic olefin copolymer portfolio, offering materials with tensile strengths up to 63 MPa, stiffness of 3.2 GPa and elongations of 3%. However, their focus has primarily been on polymer solutions rather than on integrating these materials into thermoplastic hot-melt adhesives. LS offers a notable advancement in the integration of robust mechanical properties of cyclic olefin polymers with hot-melting adhesive capabilities. Although similar to formulations in the literature, it exhibits superior lap shear strength with distinctive yielding behaviour during failure, contrasting with frequently reported sudden failure [42,51,52]. This gradual failure mechanism suggests enhanced ductility and energy absorption, potentially providing improved safety margins and stress distribution in the bonded joints. This balanced approach between COP structural integrity and HMA bonding strength opens up possibilities for high-performance adhesive applications in diverse sectors, including panel assembly, battery pack frame bonding, glass glazing and electronic component assembly.

The literature lacks fracture mechanic tests in COC or COP HMAs. This study was able to address this gap by applying established adhesive fracture tests to estimate critical energy release rates. The findings reveal a Mode I critical energy release rate of 0.41 N/mm and a Mode II of 2.57 N/mm. The resulting fracture toughness ratio between modes is 6.3, which aligns with typical values observed in paste-based adhesives [1].

Despite the LS adhesive being a development product, it is believed to have properties similar to those of cyclo-olefin-based polymers, such as excellent transparency, insulation properties and being easily dismountable. Further tests should focus on impact testing, fatigue analysis and moisture [3,55], which would allow to evaluate the long-term durability of the joints.

## 5. Conclusions

This study presents a comprehensive mechanical characterisation of a novel cyclic olefin-based polymer, a development product. The DMA test produced a $T_{g}$ of 114.2 °C but also a β-relaxation stage at 77.5 °C, which influences the mechanical performance above this temperature. The non-tacky hot-melt sheet showed typical thermoplastic behaviour under tension loading, having presented both cold drawing and strain hardening phenomenon. The results indicated a Young’s modulus of 243 MPa, a tensile strength of 18 MPa and an inferred elongation of 630%, properties comparable to similar adhesives reported in the literature. The TAST specimens presented high shear strain, up to 200%, highlighting the plastic behaviour of the thin adhesive film.

Fracture tests revealed Mode I and II critical energy release rates of 0.41 N/mm and 2.57 N/mm, respectively, resulting in a 6.3 ratio between the two. Lap shear tests outputted a strength of 8.1 MPa for the aluminium adherents and 8.5 MPa for the plasma-treated CFRP substrates, although the performance decreased at elevated temperatures near the β-relaxation stage. The failure mode was adhesive, confirmed through SEM analysis, where the film was peeled from the joint and the surface was analysed, reinforcing the absence of polymer residue on the substrate.

LS serves as a reference point in the adhesive industry because of its superior mechanical properties, including high lap shear strength and a gradual failure mechanism, rather than the brittle and sudden failure typical of other HMAs reported in the literature. Our findings indicate that this novel polymer outperforms conventional HMAs because of its unique combination of high elongation, strong adhesion and controlled failure mechanism, making it a promising candidate for advanced industrial applications. Its mechanical properties make it particularly well suited for processes where heat and pressure are applied, such as automotive panel assembly, battery pack structural bonding, glass glazing and electronic component assembly, including circuit board bonding. Future research should focus on optimising high-temperature performance and exploring behaviour under dynamic loading conditions.

## Figures and Tables

**Table 1:** Summary of mechanical properties.

Property	Units	Value
Young’s modulus, *E*	[MPa]	243.5±0.96
Yield strength, $\sigma_{y}$	[MPa]	8.2±0.4
Tensile failure strength, $\sigma_{f}$	[MPa]	18.3±1.6
Tensile failure strain, $\varepsilon_{f}$	[%]	630±70
Shear modulus, *G*	[MPa]	90 *
Shear failure strength, $\tau_{f}$	[MPa]	8.45±0.12
Shear failure strain, $\gamma_{f}$	[%]	201±23
Toughness in Mode I, $J_{I_{c}}$	[N/mm]	0.41±0.02
Toughness in Mode II, $J_{II_{c}}$	[N/mm]	2.57±0.09

* Considering a Poisson ratio of 0.4

**Table 2:** Lap shear strengths for room temperature and high-temperature tests.

Aluminium	CFRP	Plasma-Treated CFRP	Dissimilar	Aluminium (80 °C)
[MPa]	[MPa]	[MPa]	[MPa]	[MPa]
8.06±0.28	5.66±0.33	8.46±0.65	7.55±0.19	2.69±0.40

**Table 3:** Benchmark of the adhesive elastic properties.

Material	*E* [MPa]	$\sigma_{y}$ [MPa]	$\sigma_{f}$ [MPa]	ϵ [%]	$T_{g}$ [°C]	Reference
LS	243	8.24	18.3	630	114	[-]
LLDPE	193	26.4	29.9	1220	115	[39]
LLDPE + 10% COC (Topas^®^ 5013)	212	29.5	29.5	488	115	[39]
LLDPE + 30% COC (Topas^®^ 5013)	301	34.6	34.6	21	115	[39]
COC + Al powder	1500	[-]	49.5	2.5	[-]	[40]
rPO_3.1_	214	9.2	14.9	990	[-]	[42]
Polyolefin HMA	2440	6	16.8	[-]	−16	[41]
PA elastomer (36,36)	1.14	[-]	1.27	1930	4	[43]

**Table 4:** Lap shear strength benchmark and testing conditions of hot-melt adhesive.

Material	Adherend	LSS [MPa]	Test Rate [mm/min]	Failure Mode	Reference
LS	Aluminium	8.1	1	AF	[-]
EVA HMA	Stainless steel	1–4	300	Mixed	[53]
SEHMA	Aluminium	9.1	[-]	AF	[51]
PAHMA	Aluminium	3.9	5	AF	[52]
rPO_3.1_	PEEK	2.4	5	[-]	[42]

## Data Availability

The original contributions presented in this study are included in the article. Further inquiries can be directed to the corresponding author.
